# Effect of Non-Dairy Food Matrices on the Survival of Probiotic Bacteria during Storage

**DOI:** 10.3390/microorganisms5030043

**Published:** 2017-08-01

**Authors:** Min Min, Craig R. Bunt, Susan L. Mason, Grant N. Bennett, Malik A. Hussain

**Affiliations:** 1 Department of Wine, Food and Molecular Biosciences, Lincoln University, Lincoln 7647, New Zealand; Min.Min@lincolnuni.ac.nz (M.M.); Sue.Mason@lincoln.ac.nz (S.L.M.); 2 Department of Agriculture Sciences, Lincoln University, Lincoln 7647, New Zealand; Craig.Bunt@lincoln.ac.nz; 3 Department of Science and Primary Industries, Ara Institute of Canterbury, Christchurch 8140, New Zealand; Grant.Bennett@ara.ac.nz

**Keywords:** probiotic, non-dairy, survival, ambient storage condition, relative humidity

## Abstract

The viability of probiotics in non-dairy food products during storage is required to meet content criteria for probiotic products. This study investigated whether non-dairy foods could be matrices for probiotics. Selected probiotic bacteria were coated on non-dairy foods under two storage conditions, and viabilities were assessed. The non-dairy foods were coated with 5–7 log cfu g^−1^ of *Lactobacillus acidophilus* ATCC4356*^T^*, *Lactobacillus plantarum* RC30, and *Bifidobacterium longum* ATCC15707*^T^*. The coated non-dairy foods were stored at 20 °C and 20% relative humidity (RH) or 30 °C and 50% RH. Viability of probiotic bacteria was determined after 0, 2, and 4 weeks of storage. *B. longum* showed the highest survival at week 4 of 6.5–6.7 log cfu g^−1^ on wheat bran and oat, compared with 3.7–3.9 log cfu g^−1^ of *L. acidophilus* and 4.2–4.8 log cfu g^−1^ of *L. plantarum* at 20 °C 20% RH. Under the storage conditions of 30 °C 50% RH, survival of 4.5 log cfu g^−1^ of *B. longum* was also found on oat and peanut. This was two and four times higher than the population of *L. acidophilus* and *L. plantarum*, respectively. The results suggest that probiotics can survive on non-dairy foods under ambient storage conditions. However, the storage conditions, food matrices, and probiotic strains should be carefully chosen to maximize probiotic bacteria survival.

## 1. Introduction

The development of innovative food products with improved sensory properties and demonstrated health benefits is needed in the rapidly growing probiotics food sector. Probiotics are micro-organisms with demonstrated health benefits to the recipient, and are used as “food additives” in the industry. Species belonging to the genera *Lactobacillus* and *Bifidobacterium*, such as *Lactobacillus acidophilus*, *Lactobacillus plantarum*, *Lactobacillus casei*, *Bifidobacterium longum*, and *Bifidobacterium bifidum*, are considered to be probiotics with well-documented evidence [1,2,3]. To be effective, probiotics need to be alive and have a minimum population of 10^6^–10^7^ cfu per g or mL in a product [4]. However, the incorporation of probiotics into processed food products and subsequent storage can be stressful for bacterial cells, and their viability may decrease. Hence, the quality of the final probiotic product is determined by bacterial survival.

The choice of food matrix is important for the viability of probiotics during both processing and storage. Dairy-based matrices can be used to produce a range of probiotic foods [5]. There is great potential to deliver bacteria using non-dairy-based food matrices and to develop new innovative probiotic products. Examples of dairy-free probiotic foods include raw materials based on fruit, vegetable, cereal, and soy. Some meat products have also been investigated as vehicles for probiotics [6]. However, during production and storage, the survival and stability of probiotics added into fruit and/or vegetable juice as well as chocolate-coated cereal breakfast have been shown to not only depend on the food matrix, water activity, and pH of the final products, but also on the choice of probiotic species selected [7,8,9].

Most probiotic food products are recommended to be stored at or below 4 °C, resulting in higher transportation and storage costs and risking viability loss of probiotics if the storage temperatures are not properly maintained. Ambient storage conditions can expose probiotic products to many stresses—heat, acid, osmotic, and oxidative—which can decrease the survival of *Lactobacillus* and *Bifidobacteria* species in foods [10]. In order to improve the survival of probiotics in foods, some strategies have been suggested. These include adaption to sub-lethal stress, optimization of drying parameters, microencapsulation, and the addition of cell protectants, and appear to achieve suitable cell counts in the products during the processing [11,12,13]. Additionally, study of food composition, storage environment, and packaging material can assist to reveal underlying mechanisms of the survival of probiotics in the products during storage [14,15,16,17]. Besides, studying probiotic bacteria under food-like conditions will provide practical information on how to improve survival during preparation, processing, distribution, storage, and consumption [18].

This study aimed to investigate the survival of three probiotic bacteria on six non-dairy foods during storage for four weeks, and to understand the effects of food matrix, storage time, storage temperature, and relative humidity on bacterial survival.

## 2. Materials and Methods

### 2.1. Materials

Six non-dairy foods were tested in this study. Blanched peanut, rolled wholegrain oat, raisin, processed wheat bran, and desiccated coconut were purchased from the local supermarket. Extruded rice collets (produced from brown Australia medium grain rice) were manufactured by the food laboratory of the Department of Wine, Food and Molecular Biosciences at Lincoln University.

*Bifidobacterium longum* ATCC15707*^T^* and *Lactobacillus acidophilus* ATCC4356*^T^* were purchased as freeze-dried cultures from the ESR Culture Collection Centre, New Zealand. The cultures were activated in De Mann Rogosa Sharpe broth (Oxiod, Hampshire, UK) supplemented with 0.05% (*w*/*v*) l-cysteine (Sigma-Aldrich, St. Louis, MO, USA) (MRSc) at 37 °C for 20 h and 22 h for *B. longum* ATCC15707*^T^* and *L. acidophilus* ATCC4356*^T^*, respectively, under anaerobic conditions using CO_2_ generating sachets (Sigma-Aldrich, USA). One milliliter of MRSc-grown cell suspension was inoculated on a MRSc agar plate and incubated at 37 °C under anaerobic conditions for 48 h for *B. longum* ATCC15707*^T^* and for 72 h for *L. acidophilus* ATCC4356*^T^*. After that, these bacterial cells were harvested using 1 mL of a mixture of sterile glycerol and MRSc broth (30:70 *v*/*v*) and washing off the agar plate, and 0.7 mL of the blend was consequently collected in a cryovial. The cryovial was labelled and stored at −80 °C as the stock culture, or at −20 °C as the working culture.

*Lactobacillus plantarum* RC30 was isolated from cow rumen. Identification and characteristics of this isolate have been previously described by our laboratory [3]. The isolate was stored in sterile glycerol and 2X MRS broth in a ratio of 30:70 (*v*/*v*) at −20 °C as a working culture.

When required, 100 µL of thawed working culture was inoculated into 10 mL of MRSc and incubated at 37 °C under anaerobic conditions until the Optical density at 600 nm (OD_600_) value of the broth culture reached 1.8, representing populations of approximately 10^8^ cell mL^−1^. MRSc broth was also used as a blank. The culture was enumerated and transferred to 4 °C and stored for up to 4 h prior to a coating process.

### 2.2. Inoculation of Non-Dairy Food Matrices with Probiotics

Utensils were sterilized under UV light for 15 min in a level II biosafety cabinet. The six different non-dairy foods were placed in an oven at 60 °C overnight to diminish potential microbial contamination and reduce moisture. Thirty-six grams of food was weighed and placed into a 500 mL sterile Scott bottle. Then, a total of 1.8 mL of fresh culture was added into the food by transferring 0.9 mL twice. With each transfer of culture onto the non-dairy matrix, mixing and coating was achieved by rigorous manual to-and-fro shaking for three minutes at room temperature. Once prepared, the culture-coated non-dairy food was then kept in the Scott bottle in a biosafety cabinet for 3 h without a lid. After that, the material was dispensed equally into two sterile 50 mL tubes. One tube was stored at 20 °C 20% relative humidity (RH), and the other was stored at 30 °C 50% RH. All the samples were prepared in triplicate.

### 2.3. Survival of Probiotic Bacteria under Two Different Storage Conditions

Potassium acetate or magnesium nitrate was placed into a 14 L plastic container and moistened with filtered water to maintain 20% RH at 20 °C or 50% RH at 30 °C, respectively. A Thermos-Hygrometer (Tinytag ultra 2 HACH, Corby, UK) was used to monitor humidity during storage. When humidity in each container equilibrated, the samples were placed into the containers and this was regarded as zero time.

### 2.4. Enumeration of Probiotic Bacteria in the Samples

The initial loading population of *B. longum* ATCC15707*^T^*, *L. acidophilus* ATCC4356*^T^*, or *L. plantarum* RC30 on each food was determined immediately after coating. Triplicate samples (1 g) were weighed and placed in a 50 mL tube, to which 10 mL of sterile MRSc broth was added. After vortex mixing for 1 min, a serial dilution for each sample was prepared. An aliquot of 0.1 mL was plated on MRSc agar plates. Plates inoculated with *L. plantarum* RC30 and *B. longum* ATCC15707*^T^* were incubated under anaerobic conditions at 37 °C for 48 h and for 72 h for *L. acidophilus* ATCC4356*^T^*.

### 2.5. Statistical Analysis

For this study, the data was obtained from the enumeration results at each time using the analysis of variance (ANOVA) processed by a statistical analysis software (GenStat^®^, VSNi UK, Hemel Hempstead, UK). Furthermore, Fisher’s least significant difference design at a confidence level of 95% determined if there was significant difference between each treatment.

## 3. Results and Discussion

### Effects of Food Matrix and Storage Conditions on the Survival of Probiotics during Storage

Food matrix, storage conditions, and storage time significantly affected the survivability of *B. longum* ATCC15707*^T^*, *L. acidophilus* ATCC4356*^T^*, and *L. plantarum* RC30 (*p* < 0.05). The average populations of the three strains in the samples reduced with increasing storage time (Table 1). The reduction was significantly (*p* < 0.05) higher at 30 °C 50% RH compared to the storage condition of 20 °C 20% RH. Foods used as carriers had a significant effect (*p* < 0.05) on the survival of the strains on week 0, week 2, and week 4.

*B. longum* ATCC15707*^T^* was initially coated onto all substrates at about log 7 cfu g^−1^, except rice collet, which was significantly lower at approximately log 6.2 cfu g^−1^ (Table 1). *L. acidophilus* ATCC4356*^T^* and *L. plantarum* RC30 were coated onto all substrates with loads ranging from log 4.5 to log 6.3, and there was no obvious trend.

After two weeks of storage at either 20 °C 20% RH or 30 °C 50% RH, *B. longum* ATCC15707*^T^* survival reduced significantly (*p* < 0.05) when coated onto all substrates except for oat or wheat bran at 20 °C and 20% RH. After four weeks of storage at either 20 °C 20% RH or 30 °C 50% RH, only storage at 20 °C 20% RH on wheat bran did not show a significant reduction of cfu from time zero. After as little as two weeks of storage at 30 °C 50% RH, *B. longum* ATCC15707*^T^* survival on raisin had reduced to less than log 1 cfu g^−1^.

After two weeks of storage at either 20 °C 20% RH or 30 °C 50% RH, *L. acidophilus* ATCC4356*^T^* showed a significant (*p* < 0.05) reduction in survival when coated on all substrates. When *L. acidophilus* ATCC4356*^T^* was coated onto rice collet or raisin, no viable bacteria were recovered after two weeks of storage at 30 °C 50% RH.

*L. plantarum* RC30 significantly (*p* < 0.05) reduced on all substrates after two weeks of storage at 20 °C 20% RH, however it appeared to be stabilized with no further reduction for all substrates except coconut and raisin, which showed further significant (*p* < 0.05) reduction. However, there were not significant reductions of *L. plantarum* RC30 at 30 °C 50% RH at either week two or week four (*p* < 0.05). Indeed, no bacteria could be recovered after coating on raisin after 2 weeks.

Studying the survival of probiotic bacteria at 20 °C 20% RH and 30 °C 50% RH can provide useful information to evaluate the storage conditions and quality of non-refrigerated probiotic foods. We found that storage temperature and relative humidity have a significant effect on the survival of probiotic bacteria. The population of bacteria recovered from samples was higher at 20 °C 20% RH than that at 30 °C 50% RH, which is accordance with the results from other studies [19,20,21]. Viability loss of *B. lactis* BB12 at 30 °C has been shown to be related to water activity, as no viable bacteria were detected after 8 days of storage at a_w_ 0.54 compared to 0.1% of viability loss with a_w_ 0.33 after 2 weeks of storage [22]. In addition, the viability of *L. rhamnosus* GG formulated with flaxseed after storage for 14 months at 22 °C was reported to show a reduction of viability by more than 4 log cfu with a_w_ 0.43, but a slight reduction of only 0.29 log cfu with a_w_ 0.11 [23]. It has been shown that low water activity in a food carrier maintains the enzyme activity of bacteria during storage, which may contribute to improved survival [24]. While the work reported here did not measure water activity of the samples, it is reasonable to estimate that the samples produced would have equilbriated to water activities of approximatley 0.2 and 0.5 at 20 °C 20% RH or 30 °C 50% RH, respectively. Low relative humidity (and therefore low water activity) has been shown to improve survival of *L. rhamnosus* GG at 11% RH. However, this was dependent upon temperature, with less survival at 37 °C than at 25 °C [25]. It has been suggested that high storage temperature accelerates metabolic and cellular activities of probiotics, resulting in depletion of nutrients and contributing to loss of viability and the oxidation of cell contents [26]. Essentially, high temperature and high water activity (humidity) lead to reduced viability.

Under the storage condition of 20 °C 20% RH, the best survival of: *B. longum* ATCC15707*^T^* was on wheat bran; for *L. acidophilus* ATCC4356*^T^* on oat or wheat bran; and *L. plantarum* RC30 coated on oat. At storage of 30 °C 50% RH, the best survival of all three bacteria was on peanut (and oat for *L. plantarum* RC30). This is supported in the literature; peanut butter has been shown to protect probiotics such as *Streptococcus*, *Lactococcus*, *Lactobacillus*, and *Bifidobacterium* species [25]. This may be due to the buffering capacity of the fat in the peanut butter [27]. Interestingly, we only observed this under the most adverse storage conditions (30 °C 50% RH). In addition, dietary fibers may also aid the viability of probiotics; for example, oat bran increased the stability of *Lactobacillus casei* LC-1 at 10 °C, 25 °C, and 40 °C compared with inulin, unripe banana flour, and apple [28]. This may be used to explain why oat generally provided a good protection for all three bacteria at 20 °C and 30 °C in this research. Clearly, the probiotic viability when formulated with foods is linked to species and type of food matrix [22]. However, another study found that survival of *L. rhamnosus* E899 and *L. rhamnosus* E522 on oat in low pH apple juice at 20 °C was better than at 4 °C [29]. This is contrary to more frequently reported results indicating that higher storage temperature results in poorer survival.

Although the initial population in the fresh culture for each bacterium was the same at ~log 8 cfu mL^−1^, the initial loading of the bacteria on foods was different and covered a range of ~log 5 to log ~7 cfu g^−1^. Since the method of sample preparation involved direct mixing of fixed quantities of materials, it can be assumed that all bacteria were loaded onto the food carriers. Clearly, something soon after coating contributed to a reduction in cell viability. This is likely related to both the bacteria species and type of food substrate.

It is interesting to note that *B. longum* ATCC15707*^T^* was the most stable probiotic bacteria during storage in the present study, which is contrary to a general suggestion that *Bifidobacteria* are more sensitive to oxygen than *Lactobacilli* due to their anaerobic nature [4]. However, a study by Klu et al. reported that *Bifidobacterium* had the greater survival at 4 °C, 25 °C, and 37 °C during storage over 12 months compared to *Lactobacillus* and *Streptococcus* or *Lactococcus* [20]. The stability of *L. acidophilus* has been reported to be affected by nutritional status, thus affecting its cell morphology—short cells of *L. acidophilus* being more stable than long filamentous rods [15]. While subjective, our microscopic observations (data not shown) suggested more long filamentous rods and less single short rods of *L. acidophilus* ATCC4356*^T^* after anaerobic incubation for three days at 37 °C. It is possible that the *L. acidophilus* ATCC4356*^T^* samples prepared for this investigation may have contributed to their poorer stability compared to *Bifidobacteria*.

## 4. Conclusions

Storage conditions, food matrix, and species played a key role in maintaining the survival of probiotic bacteria on the six non-dairy solid foods. Loss of viability of the probiotic bacteria increased with increasing storage time, temperature, and relative humidity. Overall, wheat bran and oat are suitable food matrices to best maintain probiotic stability under the mild storage conditions of 20 °C 20% RH. Under adverse condition (30 °C 50% RH), peanut is the best matrix to maintain probiotic survival under all storage conditions. *B. longum* ATCC 15707*^T^* had the greatest survival under both storage conditions, followed by *L. plantarum* RC30 and *L. acidophilus* ATCC4356*^T^*.

In general, our research has demonstrated a new class of probiotic non-dairy food products with good shelf-life suitable for retail. Future research to assess probiotic oxidative and osmotic stress in non-dairy solid food system and packages should also be investigated to further enhance and maintain bacterial survival on non-dairy food products during a shelf-life.

## Figures and Tables

**Table 1:** Probiotic bacteria log cfu g^−1^ recovered from samples coated with *B. longum* ATCC15707*^T^*, *L. acidophilus* ATCC4356*^T^*, and *L. plantarum* RC30 as affected by food matrix, storage temperature, and humidity during four weeks.

Food Matrix	*B. longum* ATCC15707*^T^*		*L. acidophilus* ATCC4356*^T^*		*L. plantarum* RC30	
	20 °C 20% RH	30 °C 50% RH	20 °C 20% RH	30 °C 50% RH	20 °C 20% RH	30 °C 50% RH
**Rice collet**						
week 0	6.22 ± 0.10 ^jkl^	6.16 ± 0.11 ^ijk^	4.70 ± 0.07 ^klmn^	4.58 ± 0.12 ^klm^	5.10 ± 0.23 ^lmn^	4.93 ± 0.25 ^lm^
week 2	5.80 ± 0.11 ^h^	3.90 ± 0.30 ^d^	3.63 ± 0.33 ^gh^	<1 ^a^	4.01 ± 0.19 ^hij^	1.35 ± 0.20 ^c^
week 4	5.84 ± 0.21 ^g^	2.25 ± 0.20 ^c^	2.97 ± 0.20 ^de^	<1 ^a^	3.97 ± 0.02 ^hi^	<1 ^a^
**Peanut**						
week 0	7.07 ± 0.05 ^st^	7.15 ± 0.07 ^t^	5.01 ± 0.04 ^nop^	5.20 ± 0.04 ^op^	6.29 ± 0.06 ^q^	6.18 ± 0.21 ^q^
week 2	6.63 ± 0.08 ^mnopq^	5.56 ± 0.07 ^h^	3.96 ± 0.56 ^hi^	2.89 ± 0.43 ^de^	3.64 ± 0.22 ^gh^	2.25 ± 0.48 ^e^
week 4	6.53 ± 0.04 ^lmnop^	4.54 ± 0.22 ^f^	3.15 ± 0.38 ^ef^	2.00 ± 0.59 ^c^	3.57 ± 0.15 ^gh^	1.22 ± 0.43 ^c^
**Coconut**						
week 0	7.04 ± 0.01 ^rst^	7.09 ± 0.08 ^st^	4.88 ± 0.03 ^lmno^	4.90 ± 0.05 ^mno^	5.42 ± 0.22 ^no^	5.28 ± 0.20 ^lmno^
week 2	6.42 ± 0.02 ^klmn^	5.18 ± 0.05 ^g^	3.40 ± 0.18 ^fg^	2.67 ± 0.10 ^d^	2.76 ± 0.28 ^f^	1.46 ± 0.18 ^cd^
week 4	6.29 ± 0.09 ^klm^	3.58 ± 0.14 ^d^	3.20 ± 0.05 ^ef^	1.18 ± 0.33 ^b^	1.57 ± 0.14 ^cd^	<1 ^a^
**Raisin**						
week 0	7.07 ± 0.05 ^st^	7.06 ± 0.05 ^rst^	5.34 ± 0.03 ^p^	5.33 ± 0.05 ^p^	5.56 ± 0.26 ^op^	5.48 ± 0.45 ^no^
week 2	6.49 ± 0.03 ^klmno^	<1 ^a^	4.56 ± 0.08 ^kl^	<1 ^a^	2.17 ± 0.20 ^e^	<1 ^a^
week 4	5.07 ± 0.33 ^g^	0.98 ± 0.85 ^b^	2.97 ± 0.28 ^de^	<1 ^a^	0.55 ± 0.52 ^b^	<1 ^a^
**Oat**						
week 0	6.87 ± 0.10 ^pqrst^	6.98 ± 0.23 ^qrst^	4.67 ± 0.14 ^klmn^	4.49 ± 0.08 ^jk^	6.12 ± 0.33 ^q^	5.95 ± 0.26 ^pq^
week 2	6.77 ± 0.12 ^opqrs^	4.93 ± 0.15 ^g^	4.13 ± 0.09 ^i^	2.05 ± 0.18 ^c^	5.33 ± 0.08 ^lmno^	3.34 ± 0.53 ^g^
week 4	6.51 ± 0.17 ^lmno^	4.26 ± 0.32 ^ef^	3.75 ± 0.15 ^h^	0.09 ± 0.11 ^a^	4.88 ± 0.06 ^kl^	1.24 ± 0.56 ^c^
**Wheat bran**						
week 0	6.88 ± 0.44 ^pqrst^	7.02 ± 0.05 ^rst^	5.22 ± 0.14 ^op^	5.25 ± 0.01 ^p^	5.35 ± 0.39 ^mno^	5.16 ± 0.10 ^lmno^
week 2	6.96 ± 0.10 ^qrst^	5.89 ± 0.21 ^hij^	4.19 ± 0.13 ^ij^	3.97 ± 0.27 ^hi^	4.43 ± 0.40 ^jk^	1.91 ± 0.47 ^de^
week 4	6.71 ± 0.14 ^nopqr^	3.93 ± 0.21 ^de^	3.91 ± 0.04 ^hi^	1.35 ± 0.11 ^b^	4.17 ± 0.01 ^ij^	0.34 ± 0.02 ^ab^

Values are mean (±standard deviation, *n* = 3). Samples which share the same letter (a–t) for *B. longum* ATCC15707*^T^*, *L. acidophilus* ATCC4356*^T^*, or *L. plantarum* RC30 are not significantly different (*p* < 0.05).
